# Dual Roles of Ascidian Chondromodulin-1: Promoting Cell Proliferation Whilst Suppressing the Growth of Tumor Cells

**DOI:** 10.3390/md16020059

**Published:** 2018-02-11

**Authors:** Xiaoju Dou, Xiang Li, Haiyan Yu, Bo Dong

**Affiliations:** 1 Ministry of Education Key Laboratory of Marine Genetics and Breeding, College of Marine Life Sciences, Ocean University of China, Qingdao 266003, China; douxiaoju@stu.ouc.edu.cn (X.D.); lx861024@126.com (X.L.); Haiyanyu@ouc.edu.cn (H.Y.); 2 Laboratory for Marine Biology and Biotechnology, Qingdao National Laboratory for Marine Science and Technology, Qingdao 266237, China; 3 Institute of Evolution and Marine Biodiversity, Ocean University of China, Qingdao 266003, China

**Keywords:** chondromodulin-1, ascidian, cell migration, cell proliferation, angiogenesis

## Abstract

Chondromodulin-1 (ChM-1) is an extracellular matrix protein that plays crucial roles in tumor cell growth and angiogenesis in vertebrates and humans. ChM-1 is highly expressed in the invertebrate *Ciona savignyi*, a marine ascidian chosen as a model. The effect of the recombinant *Ciona* mature ChM-1 peptide (Cs-mChM-1) on cell proliferation, migration and angiogenesis was evaluated on cultured cells. The results revealed that low concentrations of Cs-mChM-1 (12.5 nM) promoted osteoblastic cell (MC3T3-E1) growth and protected cells from H_2_O_2_-induced damage. However, a higher concentration of Cs-mChM-1 (i.e., 500 nM) not only suppressed both growth and migration of tumor cells, including human cervical cancer (HeLa) cells and human neuroblastoma (SH-SY5Y) cells, but also significantly inhibited proliferation and angiogenesis of human umbilical vein endothelial cells (HUVECs). The expression levels of cyclinD1 and mitogen-activated protein kinase 1 (MAPK1) were slightly increased in Cs-mChM-1 treated MC3T3-E1 cells, whereas these genes decreased in treated HeLa cells, SH-SY5Y cells and HUVECs. This result indicates that Cs-mChM-1 modifies cell behavior by regulating cell cycle and cell adhesion. Thus, the present results reveal that recombinant peptides of ChM-1 from invertebrates can play a dual role in cell proliferation and migration of different cell types. The inhibition effects on tumor cell growth and angiogenesis indicate potential pharmaceutical applications for recombinant Cs-mChM-1.

## 1. Introduction

Chondromodulin-1 (ChM-1) is a glycoprotein originating from fetal bovine cartilage. It has a molecular mass of 25 kDa and can be converted into a 12 kDa mature peptide at the C-terminal RERR amino acid cleavage site, then subsequently secreted into the extracellular matrix (ECM) [1,2].

ChM-1 is specifically expressed in non-vascular tissues such as immature cartilage, cardiac valves, and the cornea. However, it is absent in the calcified cartilage of vertebrates [3,4]. As a chondrocyte growth factor, ChM-1 is responsible for maintaining cartilage integrity during osteogenesis [5]. ChM-1 knockout mice exhibited normal growth and fertility but abnormal osseous remodeling [3]. Moreover, recombinant human ChM-1 (rhChM-1) suppresses the proliferation of mouse splenic T cells and human peritoneal blood T cells [6]. Collectively, ChM-1 is presumed to play multiple functions in different tissues.

More importantly, rhChM-1 was demonstrated to inhibit tumor cell proliferation and tube morphogenesis in vitro [7] and prevent blood vessels growth in chick chorioallantoic membranes (CAMs) in vivo [8]. Because cell growth and new capillary sprout formation are key steps of cancer progression, ChM-1 may have a potential application in cancer therapy.

CyclinD1 and MAPK1 expression levels are considered to be important molecular markers for evaluation of the degree of tumor malignancy. Previous studies showed that the expression of cyclinD1 and MAPK1 decreased in ChM-1 transfected breast cancer cells in comparison to the transfected control cells [9]. Similarly, in rhChM-1 transfected mesenchymal stem cells, the expression level of MAPK was decreased [10]. Mera et al. (2009) also demonstrated that rhChM-1 suppresses cell proliferation by regulating protein in the tyrosine phosphatase family, which activates MAPK through rapidly accelerated threonine-protein kinase [11]. These findings indicate that ChM-1 inhibits tumor cell proliferation by suppressing cyclinD and MAPK signaling. 

Our previous work has identified that ChM-1 is highly expressed in the tunicate animal ascidian *Ciona savignyi.* As an ancestral chordate with a unique evolutionary position, ascidians are not only regarded as the link between chordates and non-chordates but also provide clues of vertebrate origin. Besides this, marine ascidians produce a variety of secondary metabolic substances, which exhibit unique biological activities [12,13]. Didemnin B, the first marine anti-tumor drug originated from the ascidian *Trididemnum solidum* [14]. Polypeptide CS5931 was identified from *C. savignyi*, and it not only inhibits cancer cell proliferation through mitochondrial-mediated apoptosis, but also represses angiogenesis by inhibiting the vascular endothelial growth factor and matrix metalloproteinases [15,16]. To date, more than 80 novel compounds (e.g., peptides, polyethers, alkaloids, prostanoids, etc.) with anti-tumor activities have been isolated from the ascidians [16,17,18,19]. Although recent studies have revealed that ChM-1 possesses beneficial effects, most of them target cartilage development or focus on its isolation or combination in vertebrates. Research on the anti-tumor effects of the highly expressed ChM-1 in *C. savignyi*, an invertebrate without cartridge or blood vessels, and its underlying mechanism of action, is still very limited.

To answer the questions above, a recombinant mature ChM-1 peptide of *C. savignyi* (refer as Cs-mChM-1) was expressed and purified, then its effects on cell behaviors were evaluated. Osteoblast precursor cell line (MC3T3-E1) is commonly used in the research of osteogenic proliferation and differentiation. Human umbilical vein endothelial cells (HUVECs) form a tube-like structure in the present of matrix. These cells are usually used to study the process of angiogenesis. Human cervical cancer (HeLa) cells and human neuroblastoma (SH-SY5Y) cells are two typical anchorage-dependent cancer cell lines, which are extensively used in proliferation and migration assays. These four cell lines were employed to evaluate the effects of recombinant Cs-mChM-1 on cell proliferation and oxidative stress restoration (MC3T3-E1), cancer cell proliferation and migration (HeLa- and SH-SY5Y cells), and angiogenesis (HUVECs), respectively.

The results in this study revealed that lower concentrations of Cs-mChM-1 promoted the growth and restored the oxidative damage of MC3T3-E1 cells, whereas higher concentrations of Cs-mChM-1 suppressed the growth and migration of HeLa cells and SH-SY5Y cells, and significantly inhibited the growth and angiogenesis of HUVECs. The findings suggest that ChM-1 from a marine ascidian plays potential roles both in antioxidant and antitumor activities.

## 2. Results

### 2.1. Acquirement of Recombinant Cs-mChM-1 

The sequence length of *Cs-mChM-1* is 333 base pairs long and encodes 110 amino acid residues. A DNA band around 300 base pairs in size was amplified (Figure S1a) by PCR and ligated into pGEX-4T vector. Then the pGEX-4T-1-mChM-1 plasmid was digested with EcoRI and BamHI to ensure the cloning and construction were correct (Figure S1b). Subsequently, the obtained plasmid was used for Cs-mChM-1 peptide expression.

The plasmid was transformed into *Escherichia coli* Rossetta (DE3), and SDS-PAGE showed that the recombinant Cs-mChM-1 was expressed in the soluble portion. The weight of recombinant peptide was found to be approximately 41 kDa, as indicated by an arrow in Figure S2a. Western blotting showed that the ChM-1 polyclonal antibody specifically bound to the target protein (Figure S2b), indicating that the Cs-mChM-1 peptide was acquired by the optimized *E. coli* Rossetta expression system.

### 2.2. Cs-mChM-1 Promoted the Growth and Restored Oxidative Damage of MC3T3-E1 Cells

**Figure 1:**
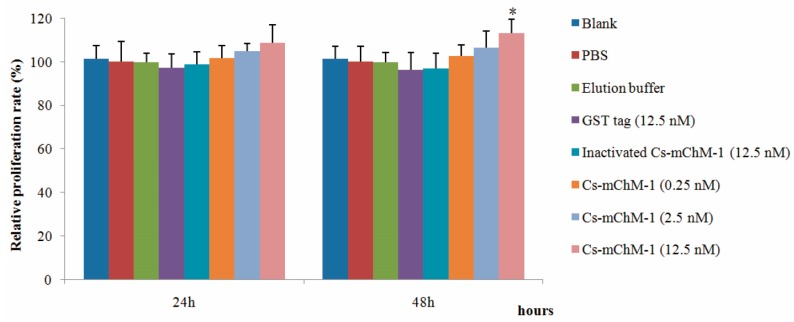
The effects of recombinant *Ciona* mature ChM-1 peptide (Cs-mChM-1) on viability of MC3T3-E1 cells. Concentrations of 0.25, 2.5, and 12.5 nM of recombinant Cs-mChM-1 were added into MC3T3-E1 cells. Cells treated with medium, phosphate buffer saline (PBS), elution buffer, 12.5 nM inactivated Cs-mChM-1, and 12.5 nM Glutathione S-transferase (GST) tag were used as controls. The relative proliferation rate of the Cs-mChM-1 treatment was significant higher than 12.5 nM inactivated Cs-mChM-1 group after 48 h. (*n* = 3, * *p* < 0.05 vs. the 12.5 nM inactivated Cs-mChM-1 group).

The effects of recombinant Cs-mChM-1 on cell growth were examined in MC3T3-E1 cells by an MTT assay. As illustrated in Figure 1, 0.25, 2.5, and 12.5 nM GST-Cs-mChM-1 treatment promoted the growth of MC3T3-E1 cells. After 48 h exposure, a 12.5 nM concentration of the recombinant peptide led to a significant increase in cell viability (*p* < 0.05). The relative proliferation rate increased by 13.21% compared with the inactivated Cs-mChM-1 group (12.5 nM) at 48 h post-treatment.

**Figure 2:**
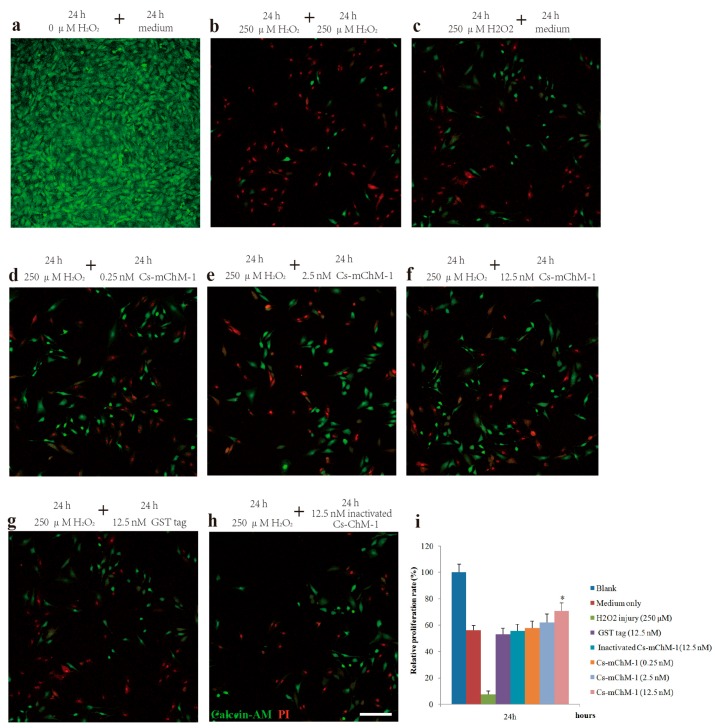
The effects of recombinant Cs-mChM-1 on the viability of MC3T3-E1 cells exposed to H_2_O_2._ (**a**) MC3T3-E1 cells were cultured in medium; (**b**) Cells were continuously exposed to H_2_O_2_ (250 μM) for 48 h; (**c**) Cells were exposed to H_2_O_2_ (250 μM) for 24 h and incubated in the medium for an additional 24 h; (**d**) Cells were exposed to H_2_O_2_ (250 μM) for 24 h followed by a 0.25 nM Cs-mChM-1 treatment for an additional 24 h; (**e**) Cells were exposed to H_2_O_2_ (250 μM) for 24 h followed by a 2.5 nM Cs-mChM-1 treatment for additional 24 h; (**f**) Cells were exposed to H_2_O_2_ (250 μM) for 24 h followed by a 12.5 nM Cs-mChM-1 treatment for an additional 24 h; (**g**) Cells were exposed to H_2_O_2_ (250 μM) for 24 h followed by a 12.5 nM GST tag treatment for an additional 24 h; (**h**) Cells were exposed to H_2_O_2_ (250 μM) for 24 h followed by a 12.5 nM inactivated Cs-mChM-1 treatment for an additional 24 h; (**i**) The activity of Cs-mChM-1 on oxidative stress cells was determined by an MTT assay (* *p* < 0.05 vs. the 12.5 nM inactivated Cs-mChM-1 treatment group). The bar represents 250 μm.

To further confirm the promotion effect, a H_2_O_2_-induced injury model was established to detect the restoration effects of Cs-mChM-1 on cell survival. The application of H_2_O_2_ induced a gradual reduction in MC3T3-E1 cell viability in a time and dose dependent manner. After 24 and 48 h of incubation, the cytotoxic dose (IC_50_) relative to the untreated group was 273.50 μM and 238.64 μM, respectively (Figure S3a,b). Therefore, a 250 μM concentration of H_2_O_2_ was used as the positive control to establish a cell damage model for all subsequent trials. As shown in Figure 2, cell growth was intensive in the negative control group (Figure 2a), while H_2_O_2_ exposure clearly induced cell apoptosis and shrinkage (Figure 2b,c). Cells incubated with Cs-mChM-1 resulted in an increase in living cells, which were stained with calcein-AM (green), and a drastic reduction in dead cells, which were stained with PI (red) (Figure 2d–f). Treatment with a 12.5 nM concentration of Cs-mChM-1 raised cell viability to 70.63%, compared with the H_2_O_2_ exposed controls (i.e., 56.02% for the medium (Figure 2c), 53.28% for the Glutathione S-transferase (GST) tag, and 55.84% for inactivated Cs-mChM-1 (Figure 2h,i)). These results showed that different concentrations of Cs-mChM-1 significantly prevented the morphological manifestations of cell damage and exhibited protective activity against H_2_O_2_-induced cell damage. This indicates that Cs-mChM-1 exerted an anti-apoptotic effect, which is generally consistent with MTT assays.

### 2.3. Cs-mChM-1 Inhibited the Proliferation of Cancer and Endothelial Cells

**Figure 3:**
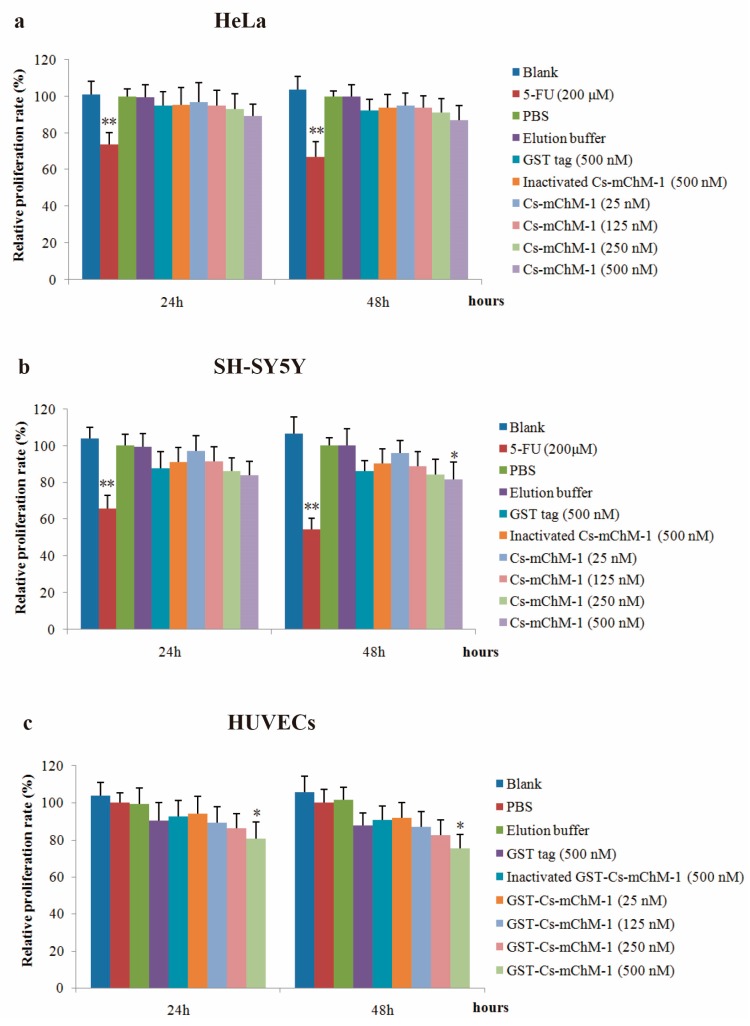
The effects of recombinant Cs-mChM-1 on the viability of cancer cells and endothelial cells i.e., (**a**) human cervical cancer (HeLa) cells; (**b**) human neuroblastoma (SH-SY5Y) cells, and (**c**) human umbilical vein endothelial cells (HUVECs). Treatments of either 25, 125, 250, and 500 nM Cs-mChM-1 were added. Cells treated with medium, 5-FU, PBS, elution buffer, 500 nM inactivated Cs-mChM-1 and 500 nM GST tag were served as control; *n* = 3, * *p* < 0.05, ** *p* < 0.01 vs. the 500 nM inactivated Cs-mChM-1 treatment group.

The effect of recombinant Cs-mChM-1 on proliferation of cancer and endothelial cells was examined. The results showed that the recombinant Cs-mChM-1 inhibited cell growth in a dose-dependent manner in HeLa cells, SH-SY5Y cells, and HUVECs. The relative proliferation rate was 87.58% in HeLa cells (Figure 3a), and 82.68% in SH-SY5Y cells (Figure 3b) after 48 h of treatment. For SH-SY5Y cells, significantly different was obtained from comparison of the 500 nM Cs-mChM-1 treatment group and inactivated Cs-mChM-1 treatment groups (500 nM) (*p* < 0.05). As for HUVECs, 500 nM concentrations of Cs-mChM-1 showed the highest levels of inhibition with 75.33% relative proliferation rate at 48 h post-treatment, which displayed a significant difference from the inactivated Cs-mChM-1 treatment group (500 nM) (*p* < 0.05) (Figure 3c).

These results indicate that 25–500 nM concentrations of Cs-mChM-1 induce cytotoxicity for HeLa cells, SH-SY5Y cells, and HUVECs.

### 2.4. Cs-mChM-1 Suppressed the Migration of Cancer Cells

**Figure 4:**
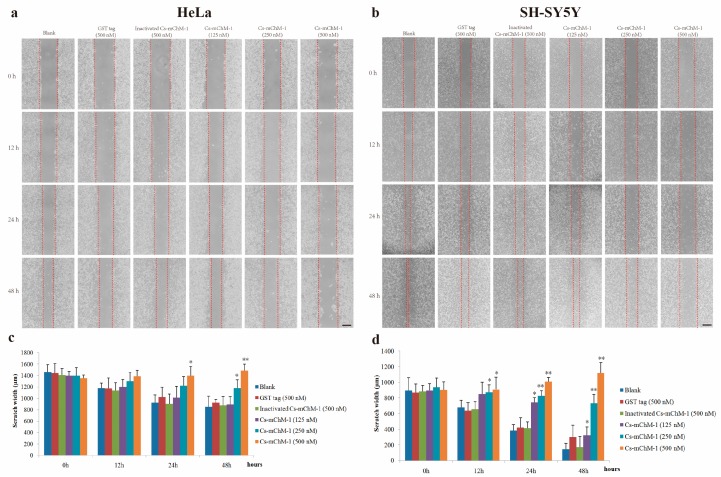
The effect of recombinant Cs-mChM-1 on HeLa and SH-SY5Y cells migration. (**a**) In HeLa cells, increased migration was obvious in blank group, while Cs-mChM-1 (125, 250, and 500 nM) treatment reduced migration capabilities; (**b**) In SH-SY5Y cells, increased migration was obvious in the blank group, while Cs-mChM-1 (125, 250, and 500 nM) treatment reduced migration capabilities; (**c**) Quantification of HeLa cell migration; (**d**) The quantification of SH-SY5Y cell migration; migration ability was determined by calculating scratch width (in μm, *n* = 3, * *p* < 0.05, ** *p* < 0.01 vs. the 500 nM inactivated Cs-mChM-1 treatment group). The bar represents 500 μm.

The migration ability of HeLa and SH-SY5Y cells were examined by a wound healing assay in the presence of Cs-mChM-1. The images taken at 0, 12, 24 and 48 h after treatment displayed significantly increased migration in the untreated group and a reduced migratory distance in the groups that received Cs-mChM-1 (125, 250 and 500 nM) treatment (Figure 4a,b). The slow tendency of migration was more obvious with prolonged time (Figure 4a,b). After incubation with 500 nM concentrations of Cs-mChM-1, the wound width of HeLa cells at 48 h was 1486.43 ± 147.99 μm. The difference was statistically significant (*p* < 0.01) compared with the 500 nM inactivated Cs-mChM-1 treatment group (894.01 ± 113.30 μm) (Figure 4c). As for SH-SY5Y cells, the wound width was 1118. 66 ± 105.73 μm for the 500 nM concentration Cs-mChM-1 treatment group, which was a significant difference (*p* < 0.01) compared with the 500 nM inactivated Cs-mChM-1 treatment group (174.55 ± 38.17 μm) (Figure 4d). These results indicate that cell invasion is suppressed by recombinant Cs-mChM-1.

### 2.5. Cs-mChM-1 Inhibited the Tube Formation of HUVECs

**Figure 5:**
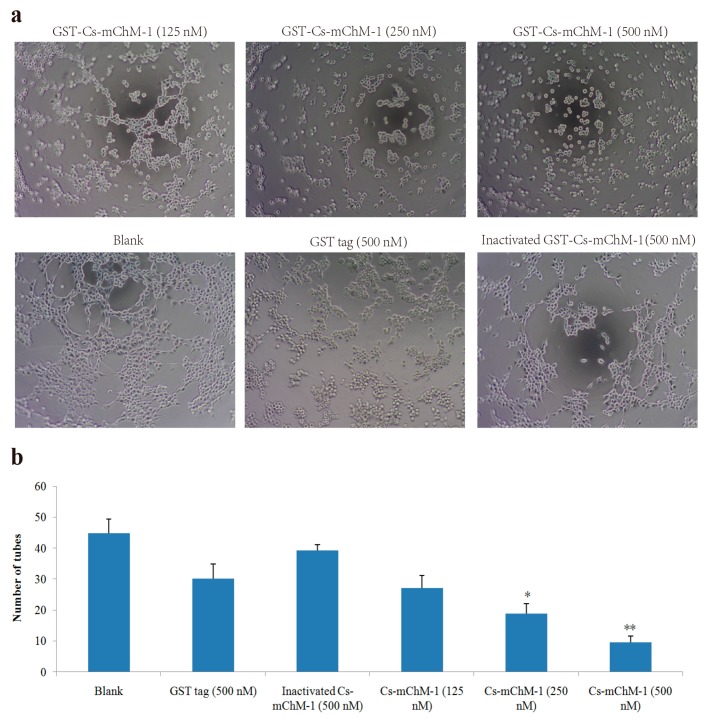
Tube formation of HUVECs in the presence of recombinant Cs-mChM-1. (**a**) Representative light microscopy images of tube formation were obtained after treatment with recombinant Cs-mChM-1, inactivated Cs-mChM-1, or GST tag; (**b**) The quantitative data of tube formation after treatment (*n* = 3, * *p* < 0.05, and ** *p* < 0.01 vs. the 500 nM inactivated Cs-mChM-1 treatment group). The bar represents 250 μm.

HUVECs could form tubule structures in 3D culture medium under the induction of structural proteins including laminin, entactin, and collagen. The effects of Cs-mChM-1 on the process of tube formation were studied. In the untreated group, HUVECs formed capillary-like tubular structures, while Cs-mChM-1 treatment showed dose-dependent inhibition of tube formation (Figure 5a). Cs-mChM-1 concentrations of 500 nM reduced tube formation to 23.1% in comparing with the 500 nM inactivated Cs-mChM-1 treatment group (Figure 5b). The above results demonstrated that Cs-mChM-1 suppressed angiogenesis by reducing tube formation in vascular endothelial cells.

### 2.6. Cs-mChM-1 Changed the Expression Level of CyclinD1 and MAPK1

**Figure 6:**
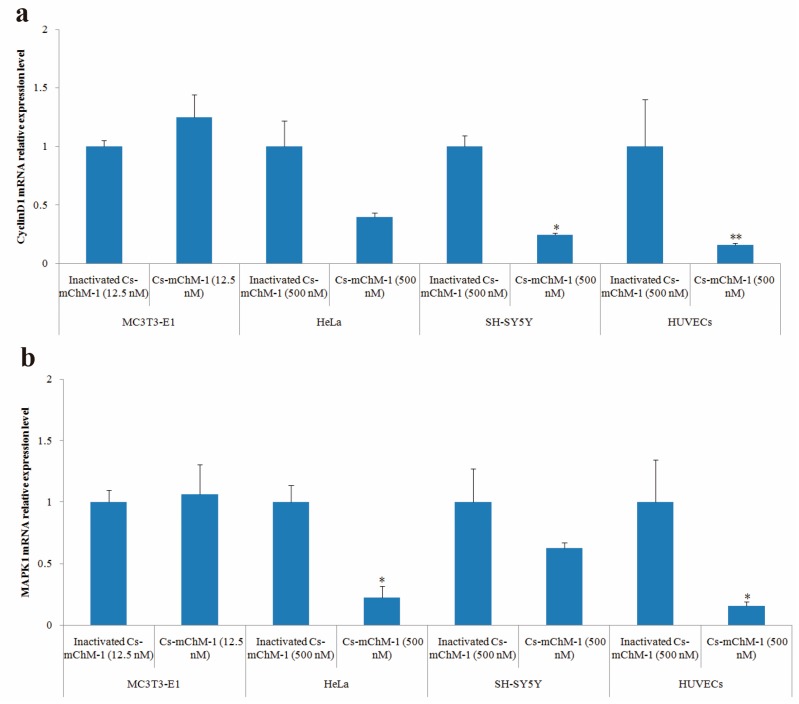
The relative mRNA expression levels determined by with qPCR after Cs-mChM-1 treatment. (**a**) cyclinD1 mRNA levels in MC3T3-E1 cells, HeLa cells, SH-SY5Y cells and HUVECs; (**b**) MAPK1 mRNA levels in MC3T3-E1 cells, HeLa cells, SH-SY5Y cells and HUVECs (*n* = 3, * *p* < 0.05, ** *p* < 0.01 vs. the 12.5 nM or 500 nM inactivated Cs-mChM-1 treatment groups).

After 48 h of treatment, cyclinD1 and MAPK1 mRNA levels in treatment groups of MC3T3-E1 cells were slightly increased but without statistical significance (*p* > 0.05), whereas the expression of cyclinD1 was significantly altered in SH-SY5Y cells (*p* < 0.05), and HUVECs (*p* < 0.01) (Figure 6a). A similar pattern was also observed in the MAPK1 expression of HeLa cells and HUVECs (Figure 6b). MAPK1 expression was decreased 3.5-fold in HeLa cells and 5.1-fold in HUVECs after treatment with 500 nM concentration of Cs-mChM-1 (*p* < 0.05). A lower concentration (12.5 nM) of Cs-mChM-1 was revealed to upregulate the expression of cyclinD1 and MAPK1 in MC3T3-E1 cells, while a higher concentration (500 nM) of Cs-mChM-1 inhibited the expression of these two genes in HeLa cells, HUVECs, and SH-SY5Y cells. This indicated that Cs-mChM-1 exerts distinct effects on the expression of cyclinD1 and MAPK1 in different cell lines.

## 3. Discussion

Cancer is the second biggest cause of mortality following cardiovascular diseases [20]. Because of the increasing prevalence of neoplasia, finding highly efficient anti-cancer compounds with low side effects is critical. The marine environment has provided a large amount of natural products [21]. Plenty of secondary metabolites identified from sessile invertebrates such as sponges, tunicates, corals, and bryozoans exhibit potent antitumor activities [22,23,24]. Nevertheless, most of these agents are available in very limited amounts, which impedes further pharmaceutical development. Under these circumstances, the recombinant Cs-mChM-1 was obtained using pGEX-4T-1-Cs-mChM-1 plasmid. The proposed protocol resulted in harvesting homogeneous Cs-mChM-1 from *C. savignyi*, and the target peptide exhibited multiple activities in different cell lines. This approach provides a foundation for future industrial production of Cs-mChM-1. 

In this study, Cs-mChM-1 was demonstrated to stimulate osteoblasts proliferation, whilst inhibit tumor cell proliferation and angiogenesis. Previous research demonstrated that both fetal-extracted ChM-1 and rhChM-1 were resistant to neovascularization from cartilage, but stimulated proteoglycan synthesis in chondrocytes [25]. ChM-1 from fetal bovine stimulated a two to three-fold increase in DNA synthesis of MC3T3-E1 cells at 1 µg/mL. The results from the cultured cells can be extended to authentic primary osteoblasts as well [26]. In contrast, High Performance Liquid Chromatography (HPLC) purified ChM-1 originating from fetal bovine inhibited DNA synthesis in BCAE cells with an IC_50_ of approximately 200 ng/mL, while 25 μg/mL of rhChM-1 suppressed DNA synthesis in HUVECs and HepG2 cells [11,27]. In line with these studies, the present data showed that Cs-mChM-1 promoted the growth of MC3T3-E1 cells while suppressing the growth and migration of SH-SY5Y cells, HeLa cells and HUVECs. DNA synthesis was increased in MC3T3-E1 cells that had undergone H_2_O_2_ oxidative injury; this was identified using the calcein-AM/PI co-staining assay.

To further reveal the functional mechanism of Cs-mChM-1, qPCR was performed to investigate the correlation between Cs-mChM-1 treatment and the expression of cell cycle markers. The results showed that regulatory gene expression in the cell cycle and DNA replication pathways were modulated by Cs-mChM-1 treatment. Cs-mChM-1 achieved a dual role probably by modulating the genes that related to G1/S phase progression.

In the current study, the expression levels of cylcinD1 and MAPK1 were examined in cell lines treated with Cs-mChM-1. CyclinD1 is a cell-cycle-related factor, which is encoded by the highly conserved cyclin family [28]. CyclinD1 interacts with the tumor suppressor protein in retinoblastomas to regulate the cell cycle [29]. Overexpressed cyclinD1 is observed frequently in a variety of tumors and contributes to tumorigenesis [30]. In this study, the present results showed that the expression of cyclinD1 was slightly upregulated in MC3T3-E1 cells but downregulated in HeLa cells, SH-SY5Y cells, and HUVECs after Cs-mChM-1 treatment. These results indicate that recombinant Cs-mChM-1 probably changes cell proliferation through the alteration of the G0/G1 cell cycle checkpoint. 

The MAPK is a family of protein kinases controlling almost all intracellular processes such as proliferation, differentiation, apoptosis, and survival through interconnected signaling cascades [10]. MAPK1 has been proven to occupy a central position in the MAPK signaling pathways and is observed to be over activated in multiple malignancies. Therefore, it is used as a potential target in oncology [31,32]. Generally, inactivation of MAPK1 decreases cell survival and promotes apoptosis and cell cycle arrest in various cell lines [33]. In the current study, Cs-mChM-1 induced the obvious downregulation of MAPK1 in HeLa cells and HUVECs in a dose-dependent manner. This result suggests that MAPK1 is involved in Cs-mChM-1-induced tumor cell suppression through altering the cell cycle.

Although the results obtained in the current research confirmed that Cs-mChM-1 promoted the proliferation of osteoblastic cell, suppressed the growth and migration of tumor cells, as well as reduced tube formation in vascular endothelial cells. It is still unclear how Cs-ChM-1 from *C. savignyi* activates intracellular signaling pathways and whether there are specific receptors for it. Obviously, further study is required to determine these mechanisms.

## 4. Materials and Methods

### 4.1. Molecular and Cell Culture Reagents

The anti-ChM-1 polyclonal antibody was administered to New Zealand white rabbits. After immunization with the ChM-1 peptide three times, the serum was collected. The antibody was then purified using affinity chromatography. A PCR Cloning Kit and T4 DNA ligases were purchased from TaKaRa Bio. Inc. (Shiga, Japan). An easy pure plasmid miniprep kit was purchased from Transgen (Beijing, China). A DNA extraction kit was purchased from GMbiolab (Taichung, Taiwan). EcoRI and BamHI were purchased from Thermo Fisher Scientific Inc. (Carlsbad, CA, USA). 5-fluorouracil (5-FU), and polymerized HRP-anti Rb IgG antibodies were purchased from Solarbio Life Science (Beijing, China). Fetal bovine serum (FBS), Dulbecco’s modified Eagle’s medium (DMEM) (high glucose), α-modified eagle’s medium (α-MEM), Roswell Park Memorial Institute (RPMI)-1640, penicillin-streptomycin and trypsin-EDTA were purchased from Invitrogen/Thermo Fisher Scientific Inc. (Frederick, MD, USA). Calcein acetoxymethyl ester (calcein-AM), propidium iodide (PI) and 3-(4,5-dimethylthiazol-2-yl)-2,5-diphenyltetrazolium bromide (MTT) were purchased from Sigma-Aldrich (St Louis, MO, USA). Basement Membrane Matrix (356234) was purchased from BD Biosciences (Bedford, MA, USA). 

### 4.2. Cells, Strains and Vector

SH-SY5Y cells were obtained from Qingdao Medical College (Qingdao, China). HeLa cells, HUVECs, and MC3T3-E1 cells were kindly provided by Dr. Liu Ya (Ocean University of China, Qingdao, China). *E. coli* Rossetta (DE3), *E. coli* DH5α, and pEGX-4T-1 vector were maintained in our lab. MC3T3-E1 cells were cultured with an α-MEM medium. HeLa cells were cultured with a RPMI-1640 medium, HUVECs and SH-SY5Y cells were cultured with DMEM medium. All cells were incubated in a medium supplemented with 10%FBS, 100 U/mL penicillin, and 100 mg/mL streptomycin, and they were maintained in 5% CO_2_ at 37 °C. Cells were sub-cultured for further experiments after they became 80–90% confluent.

### 4.3. Plasmid Construction

Fragment of cDNA encoding Cs-mChM-1 were amplified via PCR (mChM1-F: 5′-ACTGAAGATAAATTCGAAG-3′, mChM1-R: 5′-TCACATAATCACATTGGTATGAGCA-3′). PCR products and pGEX-4T-1 vectors were digested with both EcoRI and BamHI, respectively. The vectors and fragments were joined by T4 DNA ligase, and then transformed into *E. coli* Rossetta (DE3) cells. A single colony was inoculated in Luria-Bertani (LB) medium with 100 µg/mL ampicillin. The sequence of pGEX-4T-1-Cs-mChM-1 plasmid was identified using DNA sequencing (Genewiz, Suzhou, China). The plasmid construction process was illustrated in Figure S4a,b.

### 4.4. Cs-mChM-1 Expression and Peptide Purification

The second inoculation was accomplished from the initial bacterial culture. Isopropyl β-d-1-thiogalactopyranoside (IPTG) was added until absorbance reached 0.6–0.8 at 600 nm. After incubation at 20 °C for 16 h, bacteria were collected and re-suspended in PBS. It was disrupted by sonication then centrifuged. Supernatant was collected and loaded to the prepared Glutatathione Sepharose 4B column for 4 h. The column was washed out with the elution buffer (comprised of 50 mM Tris-HCl and 40 mM reduced glutathione; pH 8.0). Purified Cs-mChM-1 was examined using 12% SDS-PAGE and by Western blotting assay.

### 4.5. Cell Viability Assay

Cell viability was assessed using a MTT assay. MC3T3-E1 cells, SH-SY5Y cells, HeLa cells and HUVECs were seeded in 96-well plates at optimal densities. Cells were treated with certain concentrations of recombinant Cs-mChM-1, GST tag, or inactivated Cs-mChM-1, 5-FU (200 μM) served as a positive control. Three replicates were performed for each treatment. After 24 or 48 h of incubation, a 10 μL MTT solution (5 mg/mL) was added to each well, and plates were incubated in the dark for another 4 h. After adding 150 μL of dimethyl sulfoxide (DMSO), the optical density (OD) values of samples were analyzed at 490 nm using a multimode reader (Multiskan GO, Thermo Fisher, Waltham, MA, USA). The relative proliferation rate was calculated as follows:

Relative proliferation rate (%) = [(*A*_treated_ − *A*_blank_)/(*A*_inactivated Cs-mChM-1_ − *A*_blank_)] × 100% (1)

*A*_treated_: OD value of cells that were treated with different concentrations of Cs-mChM-1 or GST tag.

*A*_blank_: OD value of wells that contain culture medium but without cells.

*A*_inactivated Cs-mChM-1_: OD value of cells that were treated with inactivated Cs-mChM-1.

MC3T3-E1 cell oxidative stress models were established using H_2_O_2_. The toxicity of H_2_O_2_ treatment was initially assessed. Cells (5 × 10^3^ cells/well) were seeded in 96-well plates and grown for 24 h then exposed to various concentrations of H_2_O_2_ (i.e., 50, 100, 200, 300, and 400 μM) for another 24 h to obtain the IC_50_.

For the oxidative stress and restoration study, MC3T3-E1 cells were plated in 96-well plates and cultured at 37 °C for 24 h. Then a 250 μM concentration of H_2_O_2_ was subsequently added for 24 h. Fresh medium containing recombinant of Cs-mChM-1 (of concentrations 0.25, 2.5, and 12.5 nM), GST tag (12.5 nM), or inactivated Cs-mChM-1 (12.5 nM) were added to take the place of the used ones and medium. Cells were then cultured for another 24 h. Fresh medium without recombinant Cs-mChM-1 served as a control. The effects of Cs-mChM-1 on cell viability were analyzed by a MTT assay, as mentioned above.

### 4.6. Calcein-AM/PI Co-Staining Assay

Culture and treatment of MC3T3-E1 cells were done the same way as Section 4.5. After 24 h of incubation with recombinant Cs-mChM-1, cells were labeled with calcein-AM (2 μmol/L) and PI (10 μg/mL). Living cells (green cytoplasmic fluorescence) and dead cells (red nucleus fluorescence) were observed under a confocal microscope (A1R, Nikon Instruments Inc., Melville, NY, USA).

### 4.7. Wound Healing Assay

SH-SY5Y and HeLa cells were plated in six-well plates respectively. After growth to 90% confluence, wounds were made by scratching the cell surface using 10 μL pipette tips. The monolayer was washed with PBS to remove floating debris then incubated in a medium with 2% FBS containing certain concentrations of Cs-mChM-1 (i.e., 125, 250, or 500 nM), GST tag (500 nM), or inactivated Cs-mChM-1 (500 nM). Images were taken at 0, 12, 24, and 48 h after wounding. The width of the wound was estimated using ImageJ software (NIH, Bethesda, MD, USA). The migration inhibition rate was calculated with the following formula:

Inhibition rate (%) = [1 − (average width of treatment group/average width of control group)] × 100%(2)

### 4.8. Tube Formation Assay

The ability of HUVECs to form capillary-like structures after treatment was evaluated on a basement membrane (i.e., Matrigel). Ice-cold Matrigel diluted with DMEM was layered in 96-well plates and polymerized at 37 °C for 1 h. HUVECs (10^4^ cell/well) were plated onto the Matrigel layer for 4 h, then used medium was replaced with fresh medium containing Cs-mChM-1 (125, 250, 500 nM), GST tag (500 nM) or inactivated Cs-mChM-1 (500 nM). Tube formation was observed after 16 h.

### 4.9. Quantitative PCR Analysis

Total RNA was isolated using TRIzol reagent and reverse transcribed into cDNA with the cDNA Synthesis Kit (Takara, Shiga, Japan). The target gene expression was detected using the SYBR Green I Master on LightCycler^®^ 480 Real-Time PCR System (Roche, Mannheim, Germany). Expression levels relative to 18sRNA were calculated using the comparative threshold method (2^−ΔΔCt^). Primer sequences used for qPCR were listed below.

CyclinD1-F: 5′-CCGATGCCAACCTCCTCAAC-3′
CyclinD1-R: 5′-TGTTCCTCGCAGACCTCCAGC-3′
MAPK1-F: 5′-GCTGAACCACATTTTGGGTATTC-3′
MAPK1-R: 5′-CTTTGGAGTCAGCATTTGGGAAC-3′
18sRNA-F: 5′-TTTCGATGGTAGTCGCCGTGCC-3′
18sRNA-R: 5′-CCTTGGATGTGGTAGCCGTTTCT-3′

### 4.10. Statistical Analysis

All experiments were performed in triplicate. Data was presented as a mean ± SD. Statistical analyses were conducted using SPSS 17.0 software (IBM, Armonk, IL, USA). The significance levels for comparison of differences were determined with a one-way ANOVA, followed by Tukey’s HSD tests for multiple comparisons. A *p*-value less than 0.05 was regarded as statistically significant difference.

## 5. Conclusions

In summary, the present study provided evidence that Cs-mChM-1 resulted in promotion and restoration effects for MC3T3-E1 cells, whereas it reduced the proliferation and migration of cancer cell lines (SH-SY5Y- and HeLa cells), as well as inhibiting tube formation of HUVECs. The underlying mechanisms were attributed to the control of intracellular processes, including regulating the cell cycle and inactivation of the MAPK signal pathway. As a novel marine originated-peptide, Cs-mChM-1 is a potential candidate to serve as an antioxidant and antitumor agent.

## Supplementary Materials

The following are available online at http://www.mdpi.com/1660-3397/16/2/59/s1, Figure S1: Cloning and plasmid construction of Cs-mChM-1, Figure S2: Expression, purification and verification of Cs-mChM-1. Figure S3: The establishment of H_2_O_2_ oxidative injury model. Figure S4: Sequencing and construction of pGEX-4T-1-Cs-mChM-1.
