# Supplementary material for: Dual Roles of Ascidian Chondromodulin-1: Promoting Cell Proliferation Whilst Suppressing the Growth of Tumor Cells
Source: Mar Drugs. 2018 Feb 11;16(2):59. doi: 10.3390/md16020059 (PMC5852487; doi:10.3390/md16020059)
Supplement: Supplementary file 1 [file marinedrugs-16-00059-s001.pdf]

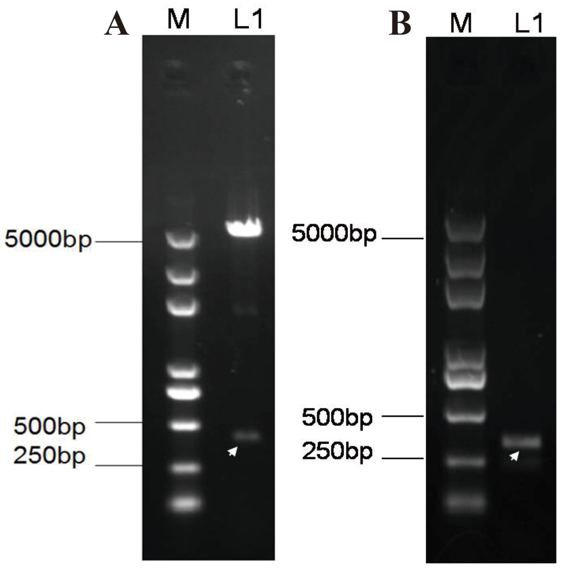

sFig. 1 Cloning and construction of Cs-mChM-1. A. Amplified mature ChM-1 fragment from *C. savignyi* cDNA, white arrows indicated mChM-1 fragment (333 bp); **B.** Digested DNA fragments of PGEX-4T-1-GST-mChM-1 with EcoRI and BamHI, white arrow showed Cs-mChM-1 fragment (333 bp) was digested off.

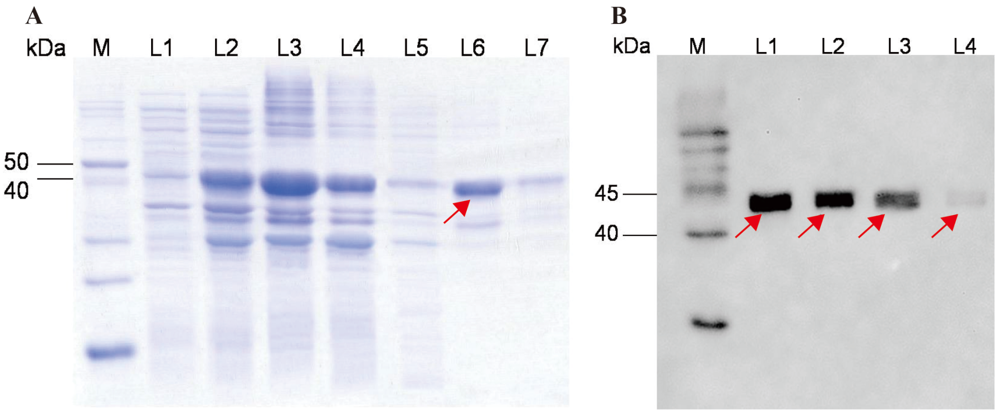

sFig.2 Expression, purification and verification of Cs-mChM-1.

A. Induction and purification of Cs-mChM-1 after optimization (M: Marker, Lane 1: protein without induction of IPTG; Lane 2: protein induced with 1mM IPTG; Lane 3: protein in precipitate after induction; Lane 4: protein in supernatant after induction; Lane 5: protein flow through GST column after binding; Lane 6: purified Cs-mChM-1; Lane 7: protein binding to beads after washing with 40 mM elution buffer. Target protein was indicated by red arrow); **B.** Western blotting of recombinant Cs-mChM-1 (M: marker; Lane1: 5 μg fusion protein; Lane2: 2.5 μg fusion protein; Lane3: 0.5 μg fusion protein; Lane4: 0.25 μg fusion protein. Target protein was indicated by red arrows).

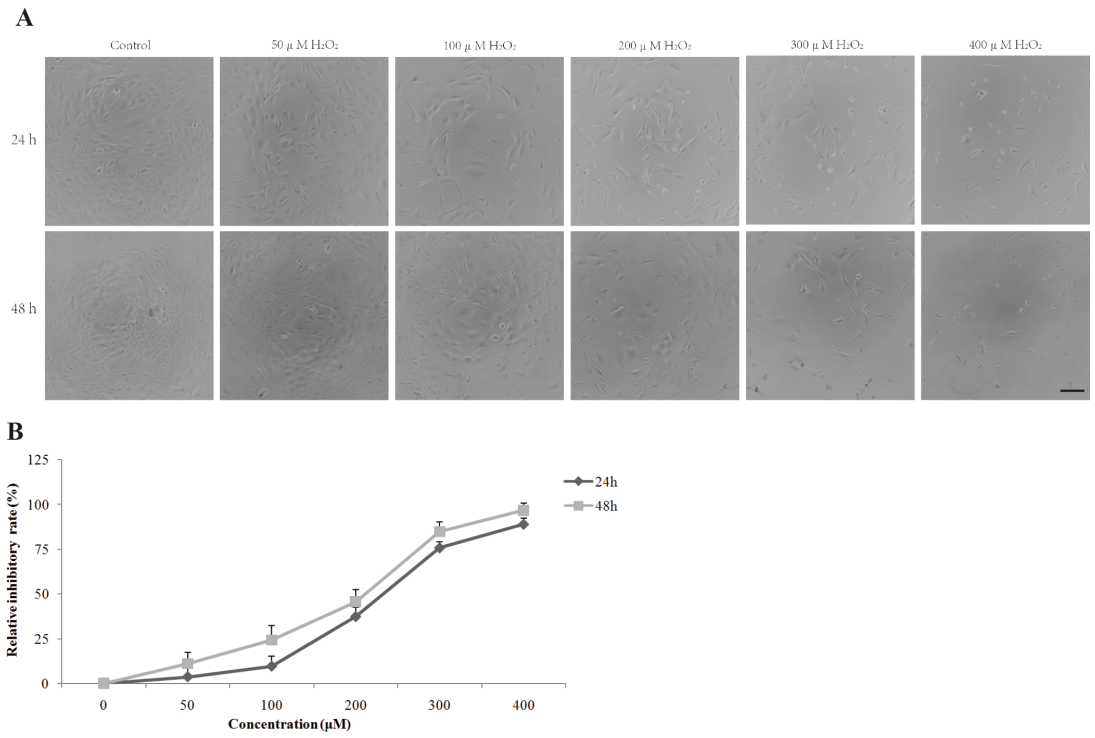

sFig.3 The establishment of H_2O_2 oxidative injury model. A. Morphology of MC3T3-E1 after H_2O_2 treatment for 24 h and 48 h; **B.** Relative inhibitory curve of MC3T3-E1 treated with H_2O_2. (n=3, The bar represents 250 μm).

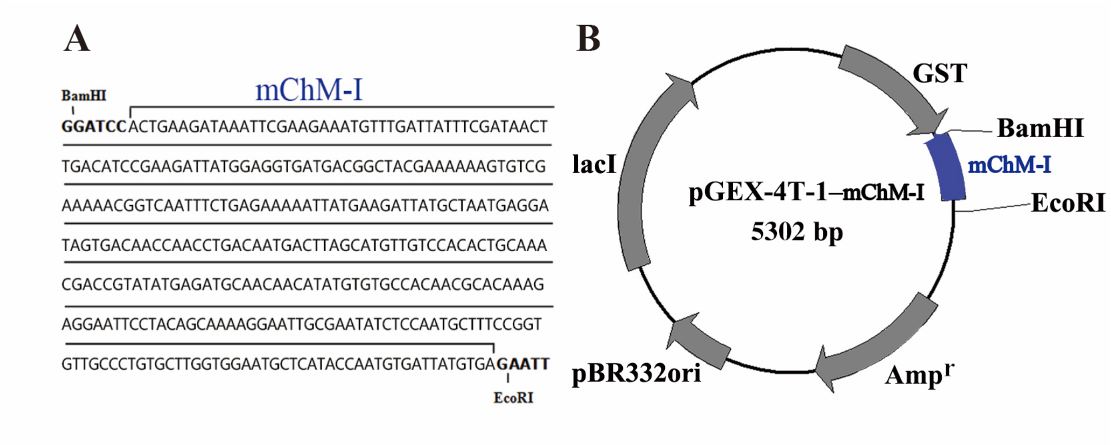

sFig.4 Sequencing and construction of pGEX-4T-1-Cs-mChM-1.

A. Overline part was the sequence of Cs-mChM-1, bold black shows digestion sites of BamHI and EcoRI; **B.** Pattern of pGEX-4T-1-Cs-mChM-1 plasmid, insertion site of Cs-mChM-1 sequence was indicated in blue.
